# Computational Study of Magnetic Particle Motion inside the Nasal Cavity under the Impact of an External Magnetic Field for Biomedical Applications

**DOI:** 10.3390/mi13111816

**Published:** 2022-10-24

**Authors:** Nikolaos Pradakis, Nikolaos Maniotis, Theodoros Samaras

**Affiliations:** 1 Department of Physics, Aristotle University of Thessaloniki, 541 24 Thessaloniki, Greece; 2 Center of Interdisciplinary Research and Innovation (CIRI), Aristotle University of Thessaloniki, Balkan Center, 570 01 Thermi, Greece

**Keywords:** computational fluid dynamics, magnetic particles, targeted drug delivery, nasal cavity, neurological diseases, magnetophoretic guidance

## Abstract

The blood–brain barrier is a highly selective semipermeable border that separates blood circulation from the brain and hinders the accumulation of substances in the central nervous system. Hence, a treatment plan aiming to combat neurodegenerative diseases may be restricted. The exploitation of the nose–brain pathway could be a promising bypass method. However, pharmaceutical uptake through the olfactory epithelium is insignificant in terms of treatment, if relying only on fluid dynamic interactions. The main reasons for this are the highly complicated geometry of the nose and the residence time of the substance. The issue can be tackled by using magnetic particles as drug carriers. With the application of an external magnetic field, further control of the particle motion can be achieved, leading to increased uptake. The present work studies this approach computationally by employing magnetite particles with a radius of 7.5 μm while a magnetic field is applied with a permanent neodymium-iron-boron magnet of $9.5\times 10^{5}\;$ A/m magnetization. Through this investigation, the best drug delivery protocol achieved a 2% delivery efficiency. The most significant advantage of this protocol is its straightforward design, which does not require complex equipment, thus rendering the protocol portable and manageable for frequent dosing or at-home administration.

## 1. Introduction

One of the most significant challenges the medical field has had to cope with during the last century are neurodegenerative diseases of the central nervous system (CNS) [1]. Given that the neural cells of the CNS do not regenerate and that their attrition is almost irreversible, it is vital to establish methods to protect and shield them as much as possible from factors that can cause permanent defects and degeneration [2]. The blood–brain barrier (BBB) constitutes the major obstacle in a treatment plan. It comprises endothelial cells, pericytes and astrocytes arranged in a way that forms a tight junction, non-permeable to most chemical substances [3]. Reportedly, more than 90% of the drugs proposed (i.e., peptides, monoclonal antibodies, protein therapeutics, etc.) as possible medicines for neurodegenerative treatment were rejected by the Food and Drug Administration (FDA) due to their inability to penetrate the BBB [4]. More than 98% of small molecules and almost 100% of macromolecules are blocked [5]. Molecules that could normally provide aid both in treatment and diagnosis turn out to be useless due to the impenetrable nature of the BBB. Factors such as molecular weight, polarity, hydrophilicity and preferability to specific carriers determine whether a substance will penetrate the BBB [6]. A very promising technique to deal with some of these restrictions is administration via the nasal route [7,8].

**Figure 1:**
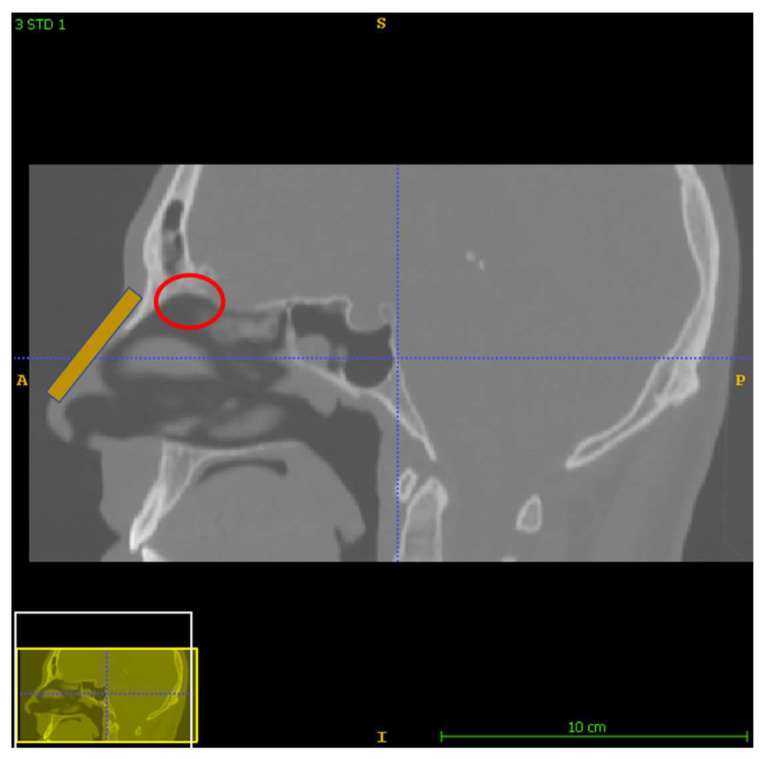
The general layout of the protocols analyzed. A permanent magnet is placed on the nasal septum (orange). The red circle indicates the olfactory region which occupies about 8% of the total nasal passage surface.

A substance can reach the brain through the nasal cavity, as evidenced by the effects of the drug cocaine [9]. Many studies have proven that the concentration of cocaine in the brain over time is greater when using the nasal route in comparison with intravenous administration, indicating the existence of a nose-to-brain path [9,10]. A substance can enter the brain using the nasal route via the olfactory epithelium, following one of three different mechanisms, i.e., using the transcellular route via the cells, using the paracellular route between the cells and, lastly, using the intracellular axonal pathway through the olfactory nerves protruding from the cribriform plate on the upper site of the nasal cavity. Nevertheless, there are also some significant limitations, such as the convoluted geometry of the nose, which limits drug deposition on the olfactory region to substantially less than 1%, hindering possible treatment plans [7,8]. Moreover, the area of the olfactory region constitutes another limitation, since it occupies only about 8% of the total surface area of the nasal route [11] (Figure 1). In addition, the metabolic mechanisms of the olfactory mucosa set a time limit on the presence of the therapeutic substances, reducing the absorption rate. The inhaled substances are blocked by the hair on the nasal vestibule or by the mucus layer covering the nasal cavity and removed through the oscillating behavior of the mucociliary clearance mechanism [9]. Through inhalation, all particles with an aerodynamic size in the range of 10–20 μm are deposited on the nasal mucosa [9]. The membrane permeability, susceptibility to degradation, as well as the site of deposition should be considered. Deposition of a substance at a different region than the olfactory epithelium can often result in entry to the blood stream and the substance being blocked from the BBB. Hence, guiding the agent while preserving its chemical properties and increasing its residence time can induce the optimum absorption conditions. According to several studies, a way to achieve this is through the use of magnetic particles as the agent carrier [11,12,13,14].

The physical properties of magnetic particles make such guidance possible through the application of an external magnetic field [15,16]. Therefore, the site of deposition does not depend only on the initial conditions and on aerodynamic interactions, but there can be a further guidance through magnetophoretic forces. Moreover, given that a particle can be covered with multiple layers, both the protection of the agent from environmental degradation, in addition to the possible toxicity of its metallic core to the nasal cavity, can be addressed [17]. Another advantage of magnetic particles is their residence time at the site of interest when applying the appropriate magnetic force [18,19]. Even though there have been computational studies exploiting magnetophoretic guidance with high efficiency delivery to the olfactory epithelium [11,12], the magnetic fields used were very high, making treatment expensive and impractical for frequent dosing or at-home administration. Additionally, such fields can only be generated using dynamic control and not through static magnets.

This study aimed in determining whether magnetophoretic guidance generated by a real permanent magnet is feasible with such low magnetic fields. In addition, we sought to investigate which magnets’ layout, among the three studied, has the best delivery efficiency. The novelty of the present work is in the thorough analysis of the optimum combination of design variables for magnetophoretic olfactory delivery, which is illustrated by the achievement two specific milestones: (1) optimization of the device design and (2) identification of the best operational variables (i.e., drug release position). As will be shown, by systematically examining both the device (magnets) and operational (release position) parameters, the olfactory delivery efficiency has been improved. In comparison with the extremely low olfactory delivery efficiency of standard nasal devices, the optimized device can deliver medications to the olfactory region at much higher doses. This study is the first to calculate the olfactory deposition efficacy for microparticles released across a line rather than a specific point of release under the influence of magnetophoretic assistance.

The structure of the text is as follows: In the methods and materials part, the nose and nasopharynx model is presented and the studied protocols are described. The mathematical background is defined by presenting the utilized approaches, namely the fluid dynamics approach, the magnetostatic approach and the particle tracing approach. Detailed information about the numerical simulation technique is also given. In the results section, the magnetic field gradient distribution and the particle trajectories are illustrated for all the protocols examined. Finally, in the Discussion section, the optimum design and best operational variables are established.

## 2. Materials and Methods

This section offers a detailed presentation of the materials and the methods implemented in the analysis. More specifically, the nose model construction, as well as the protocols of this study are described, along with the software and numerical details of the analysis. Lastly, the parametrized analysis is outlined, including the fluid dynamics, the magnetostatic and the particle tracing approaches. In this study, three different delivery approaches were analyzed based on a layout of two permanent neodymium-iron-boron (NdFeB) magnets (with a remanence of 1.2 T) placed on the nasal septum (Figure 1). The geometry was reconstructed from a real CT head image. For all protocols, the delivery efficiency was calculated on the olfactory region as the percentage of particles reaching the olfactory region of the total number of particles released in the nose.

### 2.1. The Nose and Nasopharynx Model

In order to discover the numerical solution of the problem, a numerical model based on realistic data was necessary. Thus, we used the DICOM imaging data from a male CT head scan with resolution/voxel spacing of 0.488 mm × 0.488 mm × 0.625 mm taken by embodi3D (https://www.embodi3d.com/files/file/8174-skull/) (accessed on 18 October 2018). The x axis of the reported resolution is defined as “right to left”, the y axis as “anterior to posterior” and the z axis as “inferior to superior”, according to the patient’s coordinate system. The three-dimensional array of the data image was 512 × 512 × 208, where 208 is the total number of slices in the z direction.

**Figure 2:**
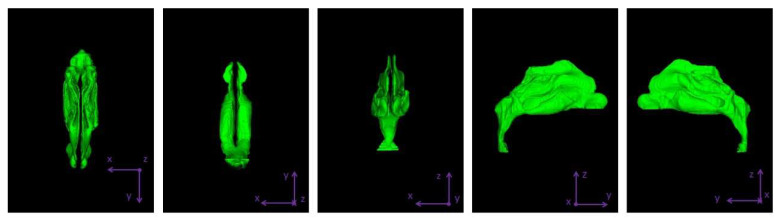
The 3D geometry of the nasal and nasopharyngeal cavities shown in various planes. The software used to segment the geometry from CT DICOM data was ITK-SNAP [20].

Since the areas of interest were the nose and the nasopharynx, the software ITK-SNAP was used to extract them [20]. The segmentation was performed semi-automatically by intervening in every CT slice in order to make sure that the resulting model was as detailed as possible. Figure 2 shows in green the segmented nasal and nasopharyngeal volumes. The sinuses were not included in the model, since they were not included in the analysis (see Section 2.2 below). The final model geometry contained 238,460 facets and was exported in STL format.

**Figure 3:**
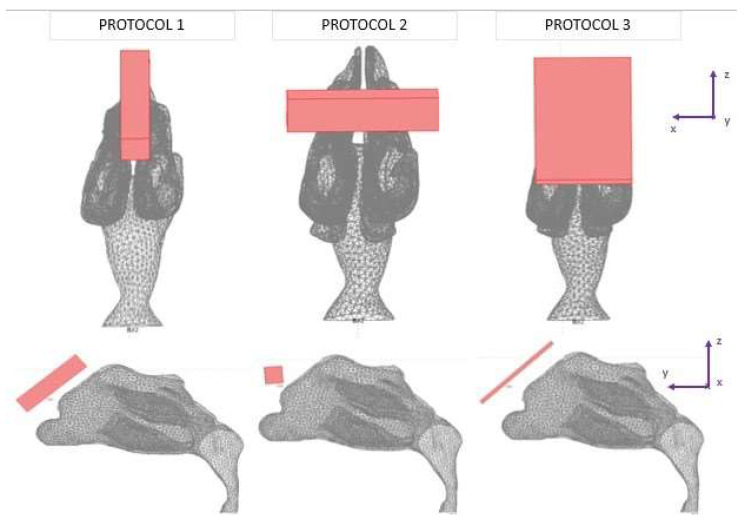
The three studied magnets’ layouts, referred to as ‘protocols’, depicted in two different reference planes. The red object denotes the magnet’s position in each layout.

In a following step, we used Autodesk Fusion 360 [21] to simplify the mesh and increase its quality to facilitate numerical solvers. The final mesh consisted of 17,484 facets. Figure 3 presents the final geometry.

Finally, the geometry was imported to COMSOL Multiphysics^®^ [22], the software which was used for the numerical solution of the problem. The final nasal volume was 2.98 × 10^−5^ m^3^ while the total surface area was 2.26 × 10^−2^ m^2^. Hence, the olfactory region surface is 8% of the total, i.e., 0.18 × 10^−2^ m^2^.

### 2.2. Studied Protocols

Three different protocols were constructed in this study, in which the tracing of the microparticles released in the nasal cavity was analyzed (Figure 3). The magnetization of the magnets used was the same in all protocols, but the geometry of the magnets varied. The layout of magnets was based on two lab magnets, so that an experimental analysis may be conducted in the future. Magnet 1 was a rectangular bar with dimensions of 1 cm × 1 cm × 4 cm. Magnet 2 was a flat rectangular magnet with dimensions of 5 cm × 3 cm × 0.2 cm.

Protocol 1 consisted of magnet 1 placed parallel to the nasal septum. The center of the magnet was placed at a 5 mm distance from the nasal cavity (Figure 3). Protocol 2 consisted of magnet 1 placed transversely on the nasal septum. It is the same as Protocol 1, but with the magnet rotated by 90 degrees (Figure 3). Protocol 3 consisted of magnet 2 placed horizontally on the nasal septum. The center of the magnet was also placed at a 5 mm distance (Figure 3).

### 2.3. The Fluid Dynamics Approach

For the airflow problem, the chemical engineering module in COMSOL was used. The flow of air inhaled was assumed to be steady, incompressible and isothermal at 36.6 °C [23]. A laminar flow was assumed, and the incompressible Navier-Stokes equations were used [24] for the stationary state. The density of air was *ρ*_air_ = 1.14 kg/m^3^, and the viscosity *η*_air_ = 1.9 × 10^−5^ Pa∙s.

There were three boundary conditions for the airflow problem:

- the inhalation steady normal inflow velocity was 0.5 m/s at the entrance of the nostril [11]; The nostrils’ total area was 1.32∙10^−4^ m^2^;
- pressure at the bottom part of the nasopharynx was set to 0 atm;
- at the inner wall (mucosa), the flow velocity was set to 0 (no-slip boundary condition).

### 2.4. The Magnetostatic Approach

For the magnetostatic problem, the AC/DC Module in COMSOL was used. For the three protocols studied, two geometrically different NdFeB magnets were used (Figure 3). The remanence magnetization value was the same for all magnets, equal to $\mu_{0}\;M=1.2$ *T*, and the vector direction was such in all protocols that the reference plane was yz (Figure 3) and the origin was the center mass point of the magnet. Specifically, the magnetic flux density component vectors for all three protocols were:

$${Β}_{x}=0\;T\;{Β}_{y}=0.771\;T\;\;{Β}_{z}=0.919\;T$$

The relative magnetic permeability of the magnets was set to $\mu_{r}=1.05$. In addition, the equations were solved in an air-filled space, where the relative magnetic permeability was considered equal to 1. The presence of tissues in this space (assumed later) did not change the value of the magnetic permeability, since human tissues do not have magnetic properties.

Magnetic insulation was assumed at the boundaries of the computational domain used for the solution of the magnetostatic problem (the domain was much larger than the nose/nasopharynx model).

### 2.5. The Particle Tracing Approach

For the particle tracing analysis, data exported from the previous approaches were used. The microparticles assumed in this study were made of magnetite. Magnetite exhibits ferromagnetic solid properties. Magnetite particles have a high magnetization, which, in combination with their low toxicity, makes them appealing for biomedical applications, compared with other magnetic materials (e.g., cobalt, chromium) [14]. The particle density was set to 5200 kg/m^3^. The microparticles were assumed to be spherical with a radius of $R_{p}=7.5\;\mu m$. The number of particles traced was set to 100, and they were released only on the left nostril to meet the limited available computational resources. The approach employed a Newtonian framework and the solution was based on ‘one-way coupling’, i.e., the continuous fluid-phase affects the particles’ motion but not vice versa. COMSOL’s software uses the Runge-Kutta approach to evaluate the particles’ path. Assuming no interaction between microparticles, the governing equation for their motion is

(1) $$\frac{d\left(m_{p}v_{p}\right)}{dt}=F_{f}+F_{m}$$

where $F_{f}$ is the drag force exerted on particles in a fluid, and is defined as

(2) $$F_{f}=-6\pi \mu R_{p}\left(v_{p}-v_{f}\right)$$

where

$v_{f}:$ speed of fluid;
$v_{p}:$ particle speed (initially, $v_{p}=0\;m/s$);
$R_{p}:$ radius of magnetic microparticle;
*μ*: dynamic viscosity of the fluid; and
$F_{m}$ is the magnetic force acting on the magnetic particles under the effect of an external magnetic field, defined as
  (3) $$F_{m}=\mu_{0}V_{p}\frac{3\chi_{p}}{\chi_{p}+3}\left(H\cdot \nabla \right)H$$
  where

$\mu_{0}:$ magnetic permeability of free space;
$V_{p}=\frac{4}{3}\pi R_{p}^{3}$: volume of the particle;
$\chi_{p}$: magnetic susceptibility; and
H: intensity of magnetic field.

Since the magnetic susceptibility of magnetite microparticles reaches generally high values, the above equation can be approximated by the following equation:

(4) $$F_{m}=3\mu_{0}V_{p}\left(H\cdot \nabla \right)H$$

Given that the microscopic motion of the particles is typically dominated by the fluid drug force, gravitational interaction was considered negligible in this approach.

### 2.6. Numerical Solutions

**Figure 4:**
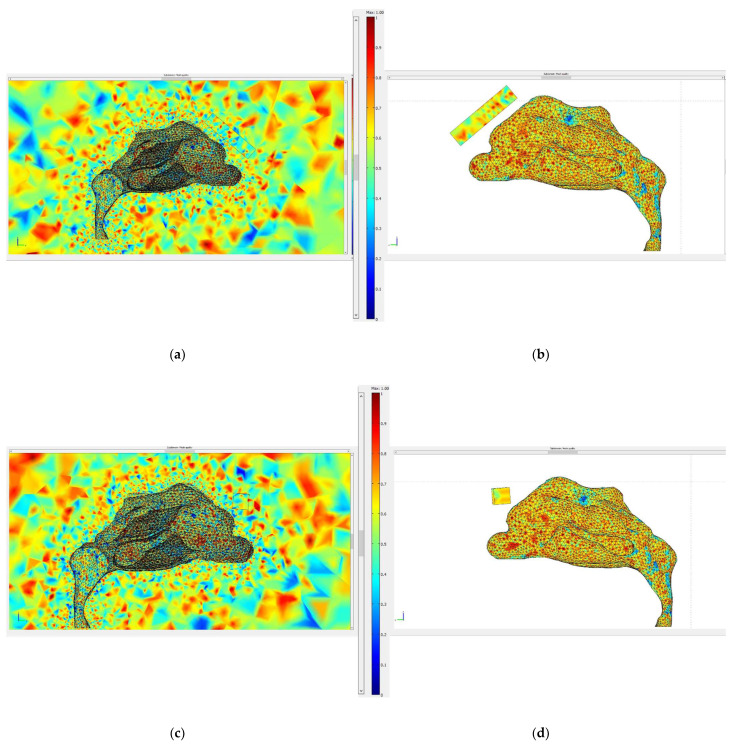

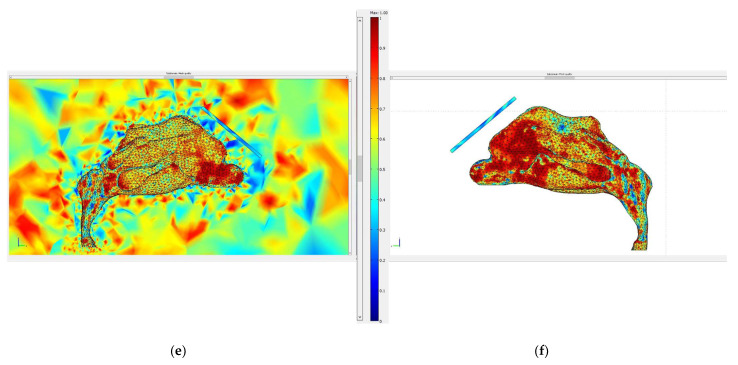
The mesh quality for the protocols in the analysis. (**a**,**b**) depict the subdomain mesh quality for protocol 1, (**c**,**d**) the subdomain mesh quality for protocol 2 and (**e**,**f**) the subdomain mesh quality for Protocol 3. Only the right half of the geometry is illustrated, i.e., the yz plane. The right-side pictures depict the mesh quality only on the domain of the nose and the magnet geometry for visual simplicity. The quality is related to the aspect ratio.

Initially, both the fluid dynamics problem, along with the magnetostatic problem, were investigated separately for the determination of the air flow and the magnetic field in space. There were three different protocols according to the magnet position, and consequently there were also three different meshes. Protocol 1 consisted of 1,393,119 elements (tetrahedral), Protocol 2 of 1,389,112 elements (tetrahedral) and Protocol 3 of 299,595 elements (tetrahedral). The element volume ration (EVR) for Protocol 1 was $6.69\times 10^{-12}.\;$ For Protocol 2 it was $\;9.33\times 10^{-12}$, while for Protocol 3 it was $3.34\times 10^{-12}.$ Figure 4 shows the mesh quality plot for the subdomain elements of nose geometry in the yz plane (Figure 3) for all protocols (COMSOL’s interface). The quality measure is related to the aspect ratio. For simplification, only the left half of the nose geometry is depicted, since we calculate the deposition efficiency only from the left nostril (see Section 2.5). The quality, q, of all the mesh elements was greater than 0.1, particularly in the region of interest (i.e., the olfactory region and vestibule), for all protocols (Figure 4), and therefore the mesh quality should not have affected the solution quality. Hence, no boundary layer mesh was added. The mesh quality for the protocols in the analysis is presented in Figure 4. Figure 4a,b depict the subdomain mesh quality for protocol 1. Figure 4c,d show the subdomain mesh quality for protocol 2, while Figure 4e,f show the same for Protocol 3. The solver used was GMRES and the preconditioner was Geometric Multigrid. The tolerance for all protocols was set to 10^−6^.

## 3. Results

In this section, the results from the particle tracing analysis for each protocol are presented. Additionally, the space gradient of the magnetic flux density B is illustrated, since it contributes to the final magnetophoretic force exerted along the paths of the microparticles.

### The Particle-Tracing Approach

Table 1 presents the final trajectories of the microparticles in the nasal cavity for the three different protocols. It also presents the approximate distance between the point of release and the nasal wall, together with the delivery efficiency of each protocol. The paths of the particles are depicted in red. The total number of particles was 100 and the release was conducted in the left nostril. The particles were released across a line in the yz plane with a length of $d_{y}\approx 1.5$ cm, engaging almost the whole length of the nostril, as depicted in Table 1. All the figures shown illustrate the yz plane. Moreover, since the gradient of the externally imposed magnetic field is a determinant of the exerted magnetophoretic force, an illustration of the gradient of B for each protocol is included in Table 2.

It is clear from the paths shown in Table 1 that the delivery efficiency is 2% for Protocol 3 and 0% for the other two protocols, since no particle is located on the olfactory region for the last two protocols (Figure 1, red circle). The results from the first protocol indicate that all the microparticles are collected in the front part of the nasal valve region, because the field gradient is larger there, close to the edge of magnet 1. Protocol 2 shows a similar behavior. There are no microparticles collected close to the olfactory region. Instead, there is a high concentration of particles on the lateral wall, just above the internal nasal valve. In contrast, Protocol 3 achieves an efficiency of 2% because it differs from the other two protocols in terms of its magnitude, the direction of its magnetic flux and its gradient.

## 4. Discussion

Magnetic particles have a wide range of applications in health care. Their capability to be guided with an external magnetic field makes them good candidates for many applications in different areas of medicine. Using magnetic particles as drug carriers to deliver drugs to the brain tissues constitutes a big challenge, since many factors should be considered. For instance, magnetic field strength and particle size should be considered in order to achieve the desired results. Magnet geometry constitutes another important factor in the delivery protocol, since the magnetophoretic force depends on the gradient of the magnetic field generated. A study on the delivery of magnetic particles to the brain via the nose-to-brain route was conducted by Xi et al. [11], who reported a delivery efficiency that was close to 0.7%, based only on aerodynamic interactions with the same inhalation velocity and particle sizes, as in the current study. However, after the application of an external magnetic field generated by a layout of four magnets located parallel to the nasal septum, with a max magnetization value of $M=8.1\times 10^{6}\;A/m$, the efficiency increased to 45%. This research also stresses the importance of the release point, which in the case of a whole-nostril-release can lead to minimum particle delivery efficiency (1.205%) under the same conditions. Another study from the same group [12] underlines the importance of the magnet strength, particle size and release point. It was an attempt to optimize their previously published analysis, and it achieved, for the same particle size of $R_{p}=7.5\;\mu m$ and the same inhalation speed of 0.5 m/s, a delivery efficiency of 67%. Nevertheless, such efficient delivery values require a dynamically controlled magnetization of $M=8.57\times 10^{6}\;A/m$ or B=10.7 T, which, in order to be produced, requires expensive and bulky equipment. Of interest are the results obtained by combining static with dynamic magnetic control. In particular, Jafari et al. [25] showed that, in heads of mice cadavers, magnetic drilling (produced by 4 A Helmholtz coils) in combination with a permanent magnet of NdFeB (0.8 T) improved the transport of magnetic nanorods (250 nm wide, 2 μm long) in the brain by 60 times compared with a static magnetic field. Nevertheless, the differentiations in both physiological and anatomical features of mice compared with humans should be considered in detail [26]. Lastly, it is important to mention that size is also important in the absorption of a substance from the olfactory bulb to the brain [27].

According to Shi et al. [28], for a particle size on the nanometer scale, much smaller than in our analysis, the delivery efficiency for a normal inhalation rate without the presence of magnetic field was approximately 0.5%. The highly complicated nasal geometry does not facilitate the accumulation of particles at the olfactory region. As shown by Kiaee et al. [29], maximum olfactory deposition averaged over all injection locations ranged from ~0.1% up to ~25% in realistic geometries. Another important conclusion of that study was that very low to no olfactory deposition was obtained for particles injected into a region approximating the lower half of the vestibule, but considerably higher olfactory deposition could be achieved for particles injected into the upper half of the vestibule. It should be pointed out that in the present study, no optimization for the point line of release (which could have improve the delivery efficiency to the olfactory region) was performed, since our aim was to show the improvement accomplished only by means of simple magnet configurations. The results collected from our analysis are in agreement with the results presented by Kiaae et al. [30] for an idealized geometry, which showed that for particles with a 15 μm diameter it was impossible to have deposition in the olfactory region. According to authors’ knowledge, this was the first attempt to estimate the olfactory deposition efficiency for microparticles released across a line instead of a specific point of release under the influence of magnetophoretic guidance. With reference to Table 1, differences can be observed among the protocols in terms of release position. This results from a trial and error method to identify the best release position in the plane for each protocol according to the magnet’s configuration. Even though the $d_{z}$ release position for the first two protocols is twice as close to the upper part of the vestibule, the magnetic configuration is such that it prevents accumulation on the olfactory region, compared with the third protocol.

**Figure 5:**
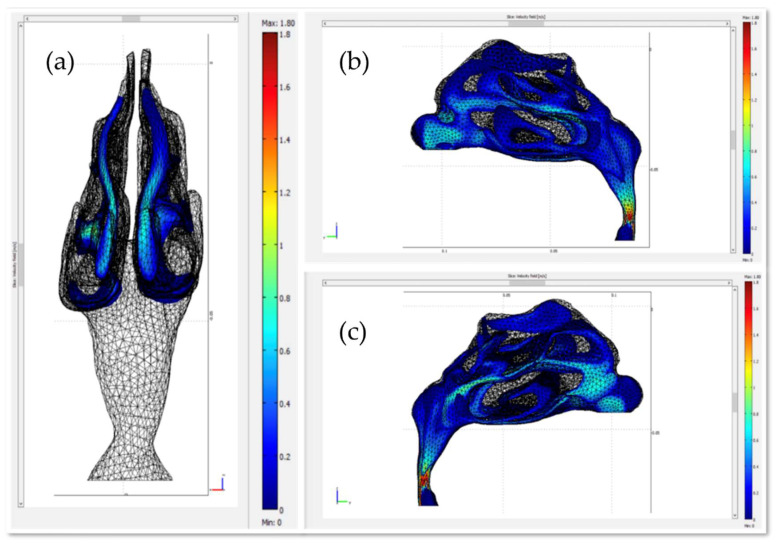
Plot of the air velocity field within the nasal cavity. (**a**) plane zx, (**b**) plane yz (**c**) plane yz rotated 180°.

Figure 5 illustrates the velocity field of air within the nasal cavity. Although the initial air velocity at the release point is 0.5 m/s, the velocity values close to the olfactory region are small (<0.2 m/s), relatively higher (~0.7 m/s) in the middle meatus, and high at the lower part of the nasopharynx (~1.5 m/s). It has been shown that for smaller particles it is better to use no or only small velocities to increase the chance of deposition at the olfactory region, otherwise they end up in the nasopharynx or farther down the upper airway [29,30]. In this study, a particle diameter of 15 μm was adopted as the size of the therapeutic agent carrier due to the high magnetic responsiveness of particles with this size [11].

When comparing the magnetophoretic guidance of a static magnetic field produced from permanent magnets with that of a dynamic control system [12], it can be seen that the delivery efficiency is greater for the latter, though it is notable that magnetophoretic guidance is also feasible through a static magnetic field. In this study, the best protocol achieved a delivery efficiency of 2% under the influence of a low-cost lab magnet with a remnant magnetic flux of 1.2 *T*. Comparing the results of [12] for similar magnetization, which in that case achieved a delivery efficiency of approximately 1%, with our analysis, it can be concluded that Protocol 3 is twice as efficient. Furthermore, compared with the work of Shi et al. [28], it can be observed that Protocol 3 has approximately four times better delivery than non-magnetic guided protocols based only on aerodynamic control. Finally, in terms of the research described in [25], no meaningful comparison can be conducted, since the geometric model parameters and the field and particle size parameters differ.

**Figure 6:**
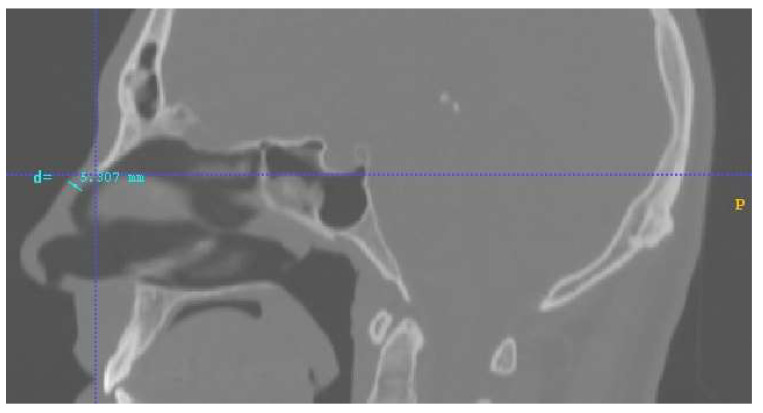
Distance from the outside nose septum to the nasal septum in human geometry. This distance of approximately 5 mm (blue line) was used as the distance of the magnets from the nasal geometric volume for the numerical analysis.

Lastly, another important factor that should be discussed and clarified is the distance of the magnets. In this research, the approximated distance of the magnets’ center of mass from the nasal septum was set at *d =* 5 mm. This value is the distance between the inner surface of the nasal septum and the outside part of the human nose septum, according to the given human geometry, on which the magnets were placed. In Figure 6, this distance is illustrated with a blue line. That is why the distance value was set at approximately equal to 5 mm. The anatomic geometry of the nose as well as the individual inter-facial differences change this distance slightly from patient to patient. Hence, taking into consideration the fact that the magnetic field is proportional to *1/d*^2^ and that the magnetic force is analogous to the magnetic field, we concluded that increasing or decreasing this distance it would strongly affect the results. Specifically, setting the magnetic layouts within the scope of a realistic distance factor *d* can exclude overestimated or underestimated values of delivery efficiency. (This could also be a possible contribution to the low efficiency achieved by the investigated protocols in comparison with similar work for similar magnetic field and particle size values [11,12]). Additionally, the maximum distance from the upper part of septum-radix to the anterior edge of the pronasale in this model was approximately 5 mm. That is the reason why the investigated magnetic layouts consisted only of one magnet and had a maximum length of 5 mm. Moreover, in our lab we possess the permanent magnets involved in the analysis, and this was an additional reason for selecting the forementioned magnets’ characteristics; that is, in order to support a possible future experimental analysis for result verification. The following table summarizes a comparison of the main contributions of our work with those reported in the literature regarding the state of the art (Table 3).

## 5. Conclusions

The BBB constitutes a tight junction that prevents many substances from entering the brain. A way to circumvent the restrictions of the BBB is using magnetic particles as drug carriers and guiding them to the site of interest through an external magnetic field. In this analysis, we studied whether magnetophoretic guidance is possible with low-cost permanent magnets placed externally on the nasal septum. The best delivery efficiency we achieved was 2%. Additionally, the proposed magnet configuration is easy to implement, low cost, and portable, and these are valuable and practical features for treatment requiring frequent dosing or at-home administration. In the future, experimental work will be needed in order to reach a final conclusion concerning the applicability and the efficiency of the herein proposed protocol.

## Figures and Tables

**Table 1:** The trajectories of the magnetite microparticles (in red) for each protocol under the influence of an external static magnetic field (1.2 *T*) are illustrated. The point of release distance from the nasal wall for each protocol is also included.

Protocols	Trajectory of Microparticles under the Impact of an External Magnetic Field	Release Distance from Nasal Wall
Protocol 1	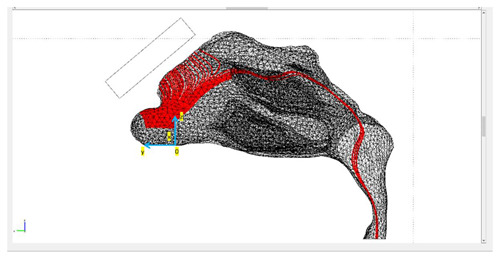	$d_{z}\approx 0.71\;\left[\mathrm{cm}\right]$ $d_{x}\approx 0.25\;\left[\mathrm{cm}\right]$ $d_{y}\approx$ nostril’s length
Protocol 2	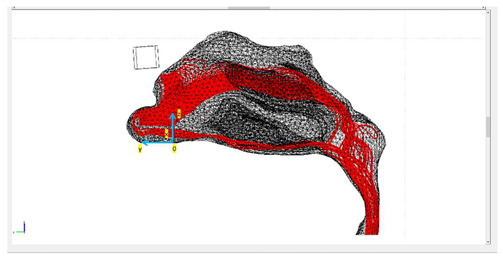	$d_{z}\approx 0.71\;\left[\mathrm{cm}\right]$ $d_{x}\approx 0.3\;\left[\mathrm{cm}\right]$ $d_{y}\approx$ nostril’s length
Protocol 3	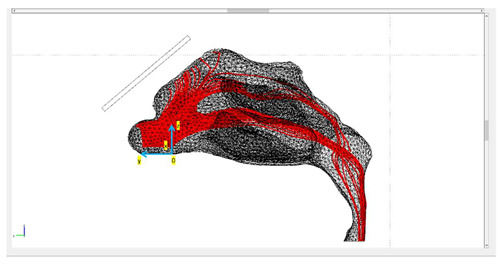	$d_{z}\approx 0.31\;\left[\mathrm{cm}\right]$ $d_{x}\approx 0.3\;\left[\mathrm{cm}\right]$ $d_{y}\approx$ nostril’s length

**Table 2:** The gradient of magnetic flux density generated by the static magnets (1.2 *T*) for each protocol.

Protocol	Gradient of the Magnetic Flux Density B Created by the Permanent Magnets (∇B)
1	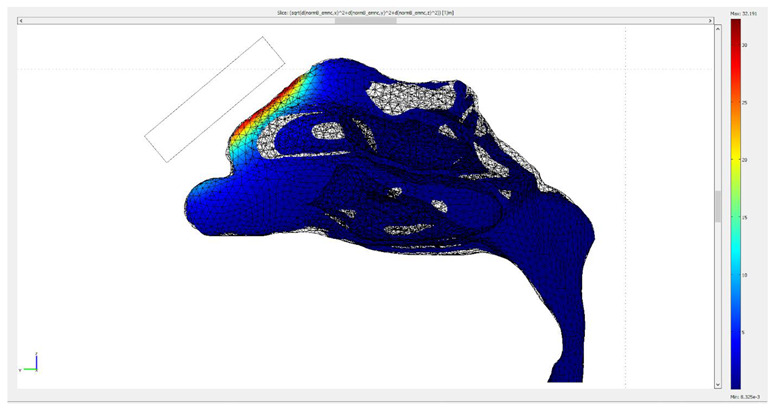
2	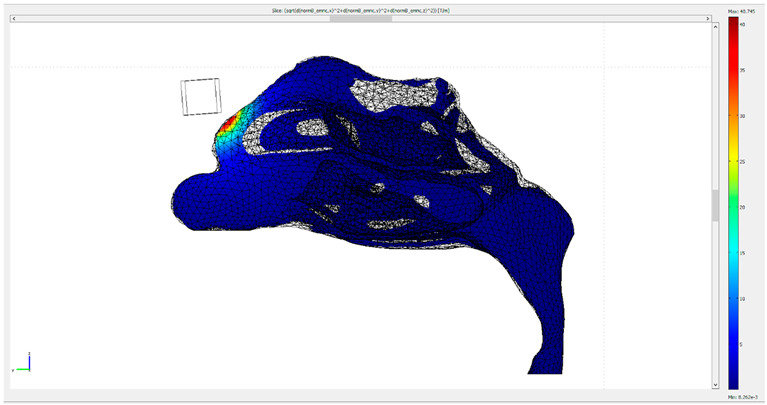
3	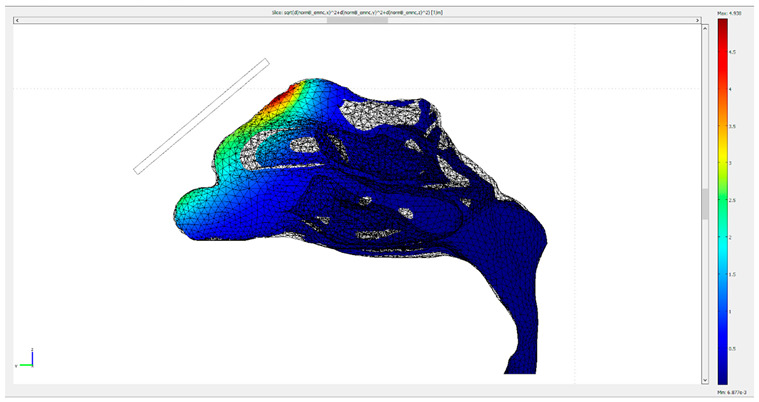

**Table 3:** Comparison of the main contributions of the work vs. those reported in the state of the art.

Reported Studies in the Literature	Main Contributions of the Present Work
The protocol for similar magnetization value ($\sim 9.5\times 10^{5}\;$ A/m) achieved a delivery efficiency of ~1% [12].	Protocol 3 is twice as efficient
The delivery efficiency for a particle size on the nanometer scale was approximately 0.5% [28].	Protocol 3 has approximately four times better delivery than non-magnetic guided protocols based only on aerodynamic control.
Specific release point of MNPs [29,30]	Calculation of the olfactory deposition efficacy for microparticles released across a line

## Data Availability

Not applicable.
